# Communication competence and behavioral challenges in breaking bad news: a single-center study of Romanian medical residents

**DOI:** 10.1186/s12909-026-08660-7

**Published:** 2026-01-24

**Authors:** Corina Mărginean, Andreea Cristina Safta, Tiberiu-Bogdan Szekely, Alexandra-Daniela Szabo, Sorin Albu, Nimród László, Corina Budin

**Affiliations:** 1 Department of Oncology and Palliative Care, Faculty of Medicine, George Emil Palade University of Medicine, Pharmacy, Science and Technology of Târgu Mureș, Târgu Mureș, Romania; 2 Mureș County Clinical Hospital, Târgu Mureș, Romania; 3 Department of Pneumology, Faculty of Medicine, George Emil Palade University of Medicine, Pharmacy, Science and Technology of Târgu Mureș, Târgu Mureș, Romania; 4 Department of Pathophysiology, Faculty of Medicine, George Emil Palade University of Medicine, Pharmacy, Science and Technology of Târgu Mureș, Târgu Mureș, Romania

**Keywords:** Breaking bad news, Behavioral preparedness, Communication skills, Medical education, Empathy, Residency training, SPIKES protocol

## Abstract

**Background:**

Breaking bad news (BBN) represents one of the most behaviorally and emotionally demanding communication skills in medical practice, particularly for residents who are still developing their professional and interpersonal competencies. Despite its ethical and psychological complexity, structured communication training remains insufficiently integrated into residency curricula in Romania.

**Methods:**

This cross-sectional, single-center study explored the behavioral preparedness, attitudes, and perceived training needs of Romanian medical residents regarding BBN. A total of 193 residents from multiple specialties at Mureș County Clinical Hospital, Târgu Mureș, completed a self-administered questionnaire assessing their experiences, emotional reactions, perceived barriers, and familiarity with communication protocols such as SPIKES. Participation was voluntary, responses were anonymous, and completion of the questionnaire implied informed consent.

**Results:**

Most respondents (94.3%) acknowledged the importance of BBN, yet only 31.6% had practical experience, and 17.6% reported formal training. Awareness and use of structured communication frameworks were limited but correlated with greater confidence and empathy. Experience increased with residency year and varied across specialties.

**Conclusions:**

Romanian medical residents recognize the ethical and emotional significance of breaking bad news but lack structured educational preparation. The findings underline the need for targeted behavioral and educational interventions to strengthen residents’ preparedness, emotional resilience, and patient-centered communication competence.

**Trial registration:**

Not applicable.

**Supplementary Information:**

The online version contains supplementary material available at 10.1186/s12909-026-08660-7.

## Background

Breaking bad news (BBN) has long been recognized as one of the most emotionally and behaviorally challenging communication skills in medical practice. Bad news has been defined as any information that seriously and adversely affects an individual’s perception of the future [1]. Such communication often triggers intense emotional responses and may alter patients’ and families’ coping mechanisms [2]. Despite its frequency, breaking bad news remains a complex interpersonal process, particularly for young physicians in training who are still developing their communication and emotional regulation skills [3].

The manner in which bad news is conveyed can have profound psychosocial consequences, influencing adaptation, trust, and treatment adherence [4]. Inadequate communication has been associated with negative psychological outcomes for patients and relatives, as well as stress, burnout, and moral distress among physicians [5]. Many doctors report fears of causing harm, being blamed, facing strong emotional reactions, or revealing their own vulnerability [6]. Organizational barriers such as time constraints, lack of privacy, and insufficient institutional support further hinder effective communication [7].

Although the importance of effective BBN is widely acknowledged, communication skills training often receives less emphasis compared to technical competencies in medical curricula. Consequently, many residents begin clinical practice insufficiently prepared for emotionally demanding interactions [3]. Inappropriate delivery of bad news by untrained physicians may undermine patient well-being and the therapeutic relationship [4].

To address these challenges, structured approaches such as the SPIKES protocol were developed, offering a six-step behavioral framework that guides clinicians through preparation, patient assessment, information sharing, empathic response, and follow-up [8]. Evidence suggests that physicians trained in SPIKES demonstrate improved confidence, empathy, and patient satisfaction [9]. Nevertheless, structured training opportunities remain limited, particularly during residency [10].

Recent international and national studies consistently highlight this deficiency: while most physicians recognize the importance of BBN, few report feeling adequately prepared [11]. Surveys of residency and fellowship program directors similarly indicate that a substantial proportion of trainees receive little or no formal instruction in breaking bad news, despite widespread agreement on the importance of such training [12]. Similar findings have been observed among medical students, who often rely on informal learning and observation rather than formalized curricula [13].

Structured approaches to breaking bad news have been increasingly incorporated into medical education through a variety of formats, including didactic lectures, small-group discussions, role-play, and simulation with standardized patients. However, evidence regarding the effectiveness of these training modalities remains heterogeneous, and relatively few controlled studies have evaluated their impact on clinical practice. Comprehensive reviews of the literature highlight both the potential benefits of such training and the methodological limitations of existing studies [14].

From a behavioral sciences perspective, understanding how residents perceive and manage the emotional and communicational dimensions of BBN is essential for designing effective educational interventions. Therefore, the present study aimed to evaluate the experience, attitudes, and training needs of Romanian medical residents regarding BBN. Conducted at the Mureș County Clinical Hospital in 2025, the study explored residents’ familiarity with structured protocols, perceived preparedness, and encountered barriers. The ultimate objective was to identify behavioral and educational gaps and to provide evidence supporting the integration of structured communication training into residency programs.

## Materials and methods

This cross-sectional, questionnaire-based study was conducted at Mureș County Clinical Hospital, Târgu Mureș, Romania, between 1 April and 31 May 2025, following approval by the Institutional Ethics Committee (approval no. 3428/04.03.2025). Participation was voluntary. All participants were provided with information regarding the study purpose, anonymity, and their right to withdraw. Completion of the questionnaire was considered to imply informed consent. A total of 193 resident physicians from medical, surgical, and emergency medicine departments were included. Eligibility criteria were active enrollment in a residency program at the hospital and willingness to complete the survey; no additional exclusion criteria were applied.

A structured and anonymous questionnaire was used to assess demographic characteristics (gender, specialty, and year of residency), previous experiences with breaking bad news, behavioral and attitudinal responses, perceived barriers, and familiarity with structured communication protocols such as SPIKES. The instrument aimed to capture both the cognitive and emotional aspects of communication preparedness in a clinical context. The questionnaire was distributed on a voluntary basis to approximately 260 medical residents across multiple departments of the hospital. Participation was open to all residents who received the invitation and wished to complete the survey. A total of 193 residents responded, corresponding to an estimated response proportion of approximately 74%.

Data were analyzed using SPSS Statistics version 26. Descriptive analyses (frequencies, percentages) were performed to summarize participants’ characteristics and responses. Associations between categorical variables were tested using Chi-square tests, while group differences were examined using the non-parametric Kruskal–Wallis H test. Statistical significance was set at *p* < 0.05. The analyses were designed to explore behavioral patterns and potential differences in preparedness across residency levels and specialties.

### Questionnaire development

The questionnaire was developed de novo for this study based on a review of the literature on breaking bad news training and communication challenges among medical residents. The instrument was designed as an exploratory, descriptive tool and included conceptually distinct items addressing perceived communication competence, emotional difficulty, training exposure, and self-reported behaviors.

## Results

### Demographic characteristics

A total of 193 medical residents participated in the study, of whom 126 (65.3%) were female and 67 (34.7%) male. The participants represented a broad range of specialties, with internal medicine being the most common (47.2%), followed by other fields (24.4%), emergency medicine (14.0%), surgical specialties (8.3%), and oncology (6.2%). The distribution by residency year was relatively balanced, ranging from the first to the sixth year of training. This demographic diversity allowed for the exploration of behavioral and educational differences across various clinical contexts and levels of experience. Detailed demographics are presented in Table 1.

**Table 1:** Demographic characteristics of the study population (*N* = 193)

Variable	Categories	n	%
**Gender**	**Male**	**67**	**34.7**
	**Female**	**126**	**65.3**
**Specialty**	**Emergency Medicine**	**27**	**14.0**
	**Oncology**	**12**	**6.2**
	**Surgical specialties**	**16**	**8.3**
	**Medical specialties**	**91**	**47.2**
	**Others**	**47**	**24.4**
**Year of residency**	**Year 1**	**42**	**21.8**
	**Year 2**	**42**	**21.8**
	**Year 3**	**31**	**16.1**
	**Year 4**	**35**	**18.1**
	**Year 5**	**42**	**21.8**
	**Year 6**	**1**	**0.5**

Mean residency experience: 2.98 ± 1.48 years; Specialty distribution mean score: 3.62 ± 1.30

### Perceptions and training

**Fig. 1:**
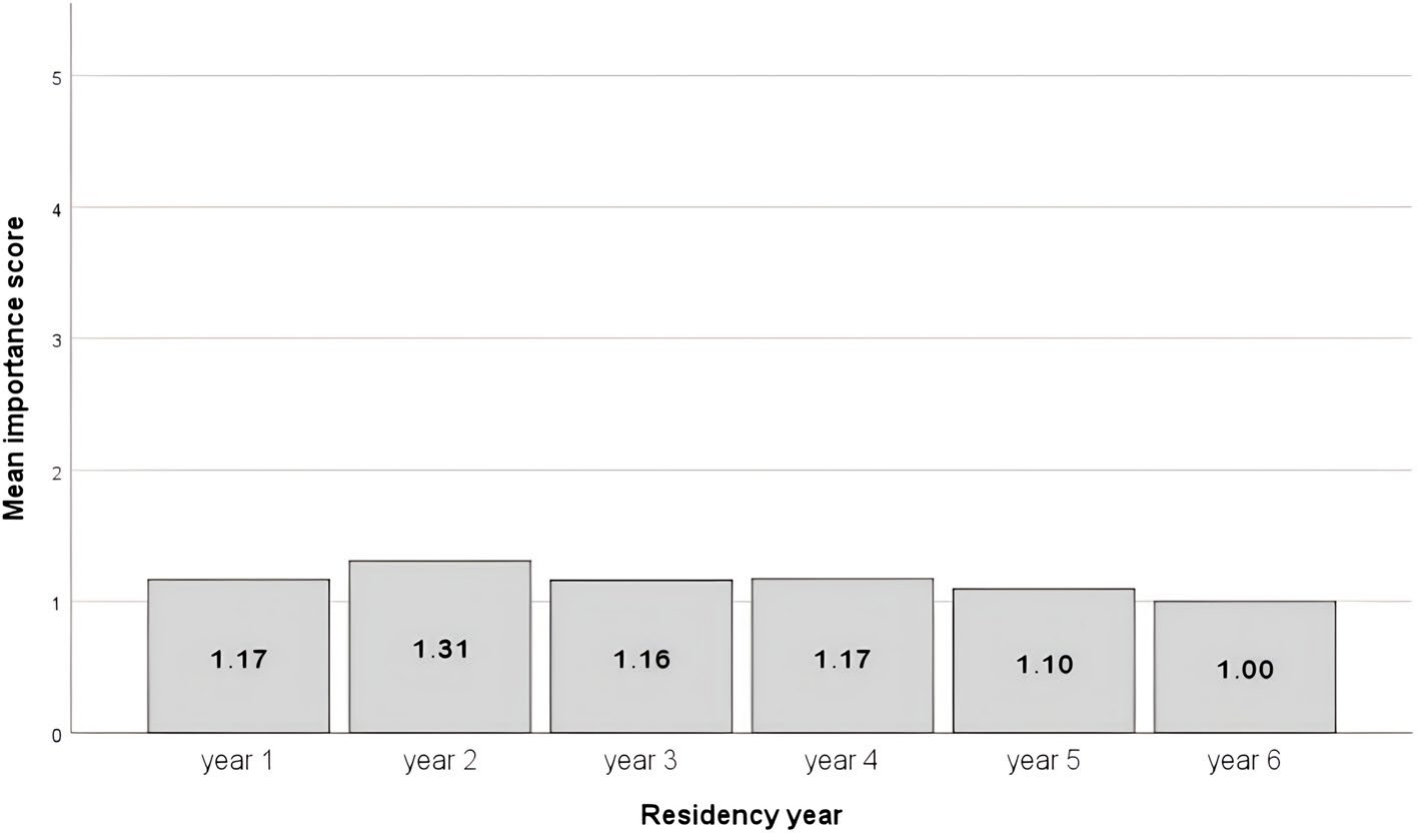
Mean importance scores for the ability to break bad news by residency year. Scores represent raw Likert-scale values (1 = very important, 5 = not important); lower scores indicate greater perceived importance. Numbers above bars indicate mean values. Sample sizes for each residency year are shown in parentheses

Most participants (94.3%) recognized the importance of effectively breaking bad news in medical practice (Fig. 1), reflecting a strong cognitive awareness of its relevance. However, only 31.6% reported having practical experience with such communication, while 68.4% indicated limited or no direct involvement, suggesting a significant gap between perceived importance and behavioral readiness. Structured communication training during residency was reported by only 17.6% of respondents, whereas 21.8% had participated in similar sessions during medical school or external workshops. These findings point to a persistent underrepresentation of formal communication training within residency programs, despite residents’ widespread acknowledgment of its value.

### Knowledge and use of protocols

Only 27.5% of participants reported familiarity with structured communication protocols, and 21.8% indicated using the SPIKES model in clinical practice. This discrepancy highlights a clear gap between theoretical awareness and behavioral implementation. The limited application of SPIKES suggests that, although residents conceptually understand the importance of structured approaches, they may lack the experiential training and emotional confidence required to apply them consistently in real interactions.

### Associations by specialty and residency year

Oncology and surgical residents rated the importance of breaking bad news significantly higher compared to other specialties (*H*(4) = 10.614, *p* = 0.031). Practical experience with BBN increased significantly with advancing residency years (*H*(5) = 26.893, *p* < 0.001), reflecting greater exposure and progressive behavioral adaptation through clinical practice. No significant differences were observed across residency years regarding knowledge or use of structured communication protocols (*p* > 0.05), suggesting that experience alone may not translate into improved protocol-based communication.

The Kruskal–Wallis analyses further indicated that self-reported experience differed significantly by both residency year (*H* = 26.893, *p* < 0.001) and specialty (*H* = 27.033, *p* < 0.001). Additionally, perceptions of who should break bad news varied across specialties (*H* = 32.332, *p* = 0.009), possibly reflecting differences in professional role expectations and emotional engagement with patients. These findings emphasize that exposure and specialty-specific culture influence behavioral preparedness more than formalized training alone. Detailed results are summarized in Table 2 (by residency year) and Table 3 (by specialty).

**Table 2:** Results of Kruskal–Wallis tests by residency year. Significant *p*-values (< 0.05) are highlighted in bold

Variable	χ^2^ (H)	df	*p*-value
Residency Year			
Importance of ability to break bad news	1.587	5	0.903
**Self-reported experience in breaking bad news**	**26.893**	**5**	**< 0.001**
Knowledge of protocols for breaking bad news	6.277	5	0.280
Use of the SPIKES protocol	2.531	5	0.772
Who should receive the bad news (perceived importance)	4.965	5	0.420

**Table 3:** Results of Kruskal–Wallis tests by specialty. Significant p-values (< 0.05) are highlighted in bold

Variable	χ^2^ (H)	df	*p*-value
Specialty			
Importance of ability to break bad news	2.318	4	0.677
**Self-reported experience in breaking bad news**	**27.033**	**5**	**< 0.001**
Knowledge of protocols for breaking bad news	6.654	4	0.155
Use of the SPIKES protocol	6.935	4	0.139
**Who should receive the bad news (perceived importance)**	**32.332**	**16**	**0.009**

## Discussion

The present study highlights a persistent gap between awareness and behavioral preparedness among medical residents regarding breaking bad news (BBN). Although most participants acknowledged its critical importance in clinical practice, only a minority had received structured education during residency or attended specialized training sessions. This discrepancy between perceived importance and applied competence mirrors previous findings indicating that physicians often view BBN as essential yet feel insufficiently prepared to manage its emotional and interpersonal demands [11].

Many residents in our sample described themselves as theoretically informed but lacking experiential confidence, reflecting earlier characterizations of BBN as an emotionally taxing skill that requires both cognitive understanding and behavioral rehearsal [1, 2]. Consistent with prior research, insufficient training appears to hinder the development of effective physician–patient–family communication and may undermine empathy and relational trust [4, 5].

Differences across specialties and training years suggest that exposure plays a key role in behavioral adaptation. Residents in surgical and oncology departments—who encounter life-altering diagnoses more frequently—reported greater awareness and confidence, likely due to repeated emotional engagement in BBN situations. However, residents in less acute specialties expressed lower preparedness, supporting the notion that experiential learning and reflective feedback are crucial for developing communication competence. Barriers such as time constraints, inadequate institutional support, and emotionally challenging settings were also recurrent, aligning with previous behavioral studies emphasizing contextual stressors in medical communication [6, 7].

Most participants expressed strong support for integrating structured communication and behavioral training modules into residency curricula. This finding reinforces the growing recognition that communication competence and emotional regulation are learnable skills rather than innate traits. Previous studies have shown that simulation-based communication skills programs using standardized patients are feasible even within resource-constrained fellowship settings and can significantly improve trainees’ self-reported comfort in breaking bad news [15]. Enhancing these competencies could mitigate distress and burnout among physicians while fostering a more empathetic and patient-centered clinical culture [3, 4].

Familiarity with the SPIKES protocol was limited, yet those who used it reported higher self-efficacy and confidence—consistent with literature showing that structured frameworks facilitate emotional containment and predictability during difficult conversations [8, 9]. Such tools not only improve communication outcomes but also strengthen physicians’ behavioral control under stress.

Our results are consistent with earlier research indicating that although structured BBN training may boost self-assurance and perceived competence, evidence of sustained behavioral change over time remains limited. The need for context-specific and well-designed interventions is highlighted by reviews of current training programs, which highlight the variation in instructional strategies and results [14].

Effective educational strategies go beyond theoretical instruction and necessitate organized opportunities for practice, feedback, and reflection, according to earlier research on breaking bad news training. Interactive techniques like role-play, small-group discussion, and simulation with standardized patients are linked to better communication skills, increased emotional awareness, and increased confidence when delivering challenging information, according to studies involving medical students and residents [13, 16, 17].

Overall, this study contributes behavioral evidence from the Romanian residency context, demonstrating that the emotional and educational challenges surrounding BBN are universal. Incorporating structured, behaviorally oriented communication training early in medical education could bridge the gap between theoretical knowledge and real-world competence, ultimately benefiting both patients and healthcare providers.

### Limitations and future directions

This study has several limitations. First, it was conducted within a single institutional setting, which may restrict the generalizability of the findings to other residency programs or cultural contexts. Second, the use of self-reported measures may introduce response and social desirability bias, as participants’ perceptions of their communication competence may differ from their observed behavioral performance. Third, the cross-sectional design limits the ability to capture developmental changes or the impact of experiential learning over time.

Future research should include multi-center and longitudinal designs to assess how communication behavior and emotional preparedness evolve throughout medical training. Experimental studies evaluating the impact of structured interventions—such as simulation-based workshops or reflective behavioral feedback—would provide stronger evidence for improving residents’ communication competence and emotional resilience. Despite these limitations, the present findings offer valuable insight into the behavioral and educational dimensions of bad news delivery within residency training.

The questionnaire was exploratory in nature and not intended as a psychometric scale. Therefore, formal validation and internal consistency testing were not performed, and findings should be interpreted descriptively.

Participation was voluntary, and the exact number of residents who received and reviewed the survey invitation could not be verified. As a result, the response proportion should be interpreted as an estimate, and self-selection and non-response bias cannot be excluded.

## Conclusions

This study demonstrated that although most medical residents recognize the importance of breaking bad news, their behavioral and emotional preparedness for this task remains limited. Only a minority reported receiving formal instruction in communication skills, and the use of structured protocols such as SPIKES was infrequent. While preparedness and confidence tended to improve with clinical experience and advancing years of residency, substantial gaps persisted across specialties and training levels.

These findings emphasize the clear need to integrate structured, behaviorally oriented communication training within residency curricula, regardless of specialty. Such programs should aim not only to enhance technical communication competence but also to strengthen empathy, emotional regulation, and self-efficacy in managing difficult conversations. By addressing these dimensions, residency education can better prepare young physicians for emotionally demanding interactions, promoting both professional well-being and a more compassionate, patient-centered healthcare culture.

## Supplementary Information

Supplementary Material 1.

## Data Availability

The data presented in this study are available on request from the corresponding author. The data are not publicly available due to privacy and ethical restrictions.

## Abbreviations

BBN: Breaking Bad News
SPIKES: Setting, Perception, Invitation, Knowledge, Emotions, Strategy
