# Supplementary material for: Communication competence and behavioral challenges in breaking bad news: a single-center study of Romanian medical residents
Source: BMC Med Educ. 2026 Jan 24;26:298. doi: 10.1186/s12909-026-08660-7 (PMC12915013; doi:10.1186/s12909-026-08660-7)
Supplement: Supplementary file 1 — Supplementary Material 1. [file 12909_2026_8660_MOESM1_ESM.docx]

Appendix 1. Questionnaire Used in the Study (English translation)

The original questionnaire was administered in Romanian.

It aimed to assess residents' experience, attitudes and training needs in breaking bad news.

**Participant Information and Consent**
You are invited to participate in a research study exploring medical residents’ experiences with breaking bad news. Participation is voluntary, responses are anonymous, and no identifiable data will be collected. You may choose not to answer any question or withdraw at any time before submission. By completing this questionnaire, you indicate your informed consent to participate.

Section I – Socio-demographic data

1. Gender *

□ Female

□ Male

2. Medical specialty

□ Emergency Medicine

□ Oncology

□ Surgical specialties

□ Medical specialties

□ Other: ___________

3. Level of clinical experience (residency year)

□ 1st year resident

□ 2nd year resident

□ 3rd year resident

□ 4th year resident

□ 5th year resident

□ 6th year resident

Section II – Residents’ experiences and attitudes regarding breaking bad news

4. How important do you consider the ability to break bad news?

□ Very important

□ Important

□ Moderately important

□ Slightly important

□ Not important

5. Do you consider that you have experience in breaking bad news?

□ Yes

□ No

6. Are you familiar with protocols for breaking bad news?

□ Yes

□ No

7. Do you use the SPIKES protocol when breaking bad news?

□ Yes

□ No

8. Who do you consider it most important to inform when delivering bad news?

□ The patient

□ The family

□ Both the patient and the family together

9. Which of the following methods do you consider most appropriate for delivering bad news? (Multiple answers allowed)

□ Email

□ Telephone

□ Face-to-face conversation

□ Written communication / letter

□ Other methods (depending on context) – please specify

10. If “Other methods” selected, please specify:

11. How long had you known the patient before delivering bad news?

□ A few hours

schi□ A few days

□ A few weeks

□ More than one month

12. How often do you deliver bad news in your clinical activity?

□ Daily

□ Weekly

□ Sometimes

□ Rarely

□ Never

13. Does your institution have a protocol for breaking bad news?

□ Yes

□ No

□ I don’t know

14. Do you believe that both the patient and their family have the right to be informed about bad news?

□ Yes

□ No

15. If yes, how do you think the communication should be approached? (Multiple answers allowed)

□ Allow the patient to decide what details they wish to receive about the bad news

□ Allow the patient to decide whether bad news may be disclosed to their family

□ Present all treatment or care options and associated risks

□ Offer the patient time to reflect before making decisions and schedule a follow-up meeting

16. What do you consider to be communication barriers in breaking bad news? (Multiple answers allowed)

□ Lack of skills in breaking bad news

□ Fear of causing negative emotions in the patient and their reactions

□ Fear of the family’s reaction

□ Fear of one’s own emotional distress

□ Lack of time and appropriate setting

□ Other reason – please specify

17. If “Other reason” selected, please specify:

18. What type of bad news have you delivered? (Multiple answers allowed)

□ Patient death

□ Cancer diagnosis

□ Progressive chronic disease diagnosis

□ Treatment or surgical failure

□ Communication of unfavorable prognosis

19. How do you prepare for breaking bad news? (Multiple answers allowed)

□ I adapt my language to the patient’s level of understanding

□ I study the patient’s medical record in detail (diagnosis, treatment plan, side effects, etc.)

□ I reflect on how to offer emotional support to the patient

□ I discuss with the healthcare team to identify the best communication approach

Section III – Assessment of the need for specific training to improve communication skills in breaking bad news

20. How would you evaluate your current level of preparedness in breaking bad news?

□ Not prepared at all

□ Very little prepared

□ Theoretically prepared but without practical experience

□ Theoretically prepared and with practical experience

21. Have you received any training on breaking bad news during your residency rotations?

□ Yes

□ No

22. Have you attended any specific training programs related to breaking bad news?

□ Yes

□ No

23. If yes, when did you attend such programs?

□ During medical school

□ During residency curriculum

□ Through postgraduate professional courses

□ Private communication courses

24. Have you ever been supervised while breaking bad news?

□ Yes

□ No

25. Who supervised you when you communicated bad news? (Multiple answers allowed)

□ A senior resident

□ A specialist physician in your field

□ A faculty member involved in your residency training

□ A hospital psychologist

□ I was not supervised

26. Did you receive feedback from the person who supervised you?

□ Yes

□ No

27. Which methods do you consider most effective for improving performance in breaking bad news? (Multiple answers allowed)

□ Direct observation of experienced medical staff delivering bad news

□ Personal experience combined with feedback from a supervisor

□ Participation in continuing education courses on communication of bad news

□ Other methods (e.g. online references, etc.)

28. If “Other methods” selected, please specify:

29. Do you think a structured module on breaking bad news should be included in residency training?

□ Yes

□ No
